# *In silico* Design of Novel Histone Deacetylase 4 Inhibitors: Design Guidelines for Improved Binding Affinity

**DOI:** 10.3390/ijms21010219

**Published:** 2019-12-28

**Authors:** Shana V. Stoddard, Kyra Dodson, Kamesha Adams, Davita L. Watkins

**Affiliations:** 1 Department of Chemistry, Natural Sciences Division, Rhodes College, 2000 North Parkway, Memphis, TN 38112, USA; 2 Department of Chemistry and Biochemistry, College of Libera Arts, University of Mississippi, P.O. Box 1848, Oxford, MS 38677, USA; 3 Department of Chemistry, Division of Natural and Mathematical Sciences, LeMoyne-Owen College, 807 Walker Avenue, Memphis, TN 38126, USA

**Keywords:** HDAC, molecular docking, inhibitor design, molecular interactions, glioma, HDAC4

## Abstract

Histone deacetylases (HDAC) are being targeted for a number of diseases such as cancer, inflammatory disease, and neurological disorders. Within this family of 18 isozymes, HDAC4 is a prime target for glioma, one of the most aggressive brain tumors reported. Thus, the development of HDAC4 inhibitors could present a novel therapeutic route for glioma. In this work, molecular docking studies on cyclopropane hydroxamic acid derivatives identified five novel molecular interactions to the HDAC4 receptor that could be harnessed to enhance inhibitor binding. Thus, design guidelines for the optimization of potent HDAC4 inhibitors were developed which can be utilized to further the development of HDAC4 inhibitors. Using the developed guidelines, eleven novel cyclopropane hydroxamic acid derivatives were designed that outcompeted all original cyclopropane hydroxamic acids HDAC4 inhibitors studied *in silico*. The results of this work will be an asset to paving the way for further design and optimization of novel potent HDAC4 inhibitors for gliomas.

## 1. Introduction

Glioma tumors represent 80% of primary malignant brain tumors. There have been significant challenges in developing targeted glioma therapeutics to improve the poor prognosis associated with this tumor type [1]. Thus, new directions are needed to advance the therapeutic options for glioma. It is known that many tumor types have variable expression of histone deacetylases (HDAC), a family of 18 isozymes known to be associated with regulation of gene expression, DNA repair, and stress response [2,3,4,5,6]. Glioma, the most deadly brain tumor has recently been shown to overexpress histone deacetylase 4 (HDAC4). The upregulation of HDAC4 in glioma cells supports tumorigenesis [7]. Thus, inhibition of HDAC4 may present an option to develop a targeted therapeutic for glioma. HDAC4, which is a class II HDAC Zn^2+^ dependent enzyme [8,9] has also been shown to be an important target for other neurological disorders [10,11,12] and other cancer types as well [13,14,15]. Due to HDAC4 pathogenic function in glioma and several other cancerous disorders, this receptor serves as an ideal target for therapeutic design.

Class II HDAC is subdivided into Class IIa and Class IIb: HDAC4 is classified as Class IIa. Previous work on the development of Class IIa selective HDAC inhibitors has led to the discovery of trifluromethylketones and cyclopropane hydroxamate acid derivatives [16,17,18,19]. The hydroxamate family of compounds has been pursued in the development of therapeutics for Huntington’s disease (HD), which is an incurable and progressive neurodegenerative disorder known to be responsive to a reduction in HDAC4 levels [16,20] but has not currently been pursued as a method for glioma therapy. Some of the trifluoromethyl ketone HDAC inhibitor derivatives developed were not shown to have decent stability [18], however, some work has been done to improve these challenges [19]. Prior work on the development of the hydroxamic acid class of HDAC4 inhibitors has focused significantly around the exploration of heteroaromatic capping moieties [16], the addition of a fluorine atom on the cyclopropane ring for enhanced binding [21], and the positioning of polar groups on the lower selectivity pocket of HDAC4 [22]. More work can be done to expand our knowledge of optimization strategies for HDAC4 inhibitors.

The current study was undertaken to develop optimization guidelines for HDAC4 inhibitors in the hydroxamic acid class. Evaluation of additional structural details that could be exploited to enhance the binding affinity of HDAC4-inhibitors would continue further research in the area. Thus, in this work, in silico analysis of molecular interactions that would promote binding of HDAC4-inhibitors has been performed. Identification of novel interactions leading to the enhanced binding ability of cyclopropane hydroxamic acid HDAC4-inhibitors was then utilized as a design template leading to 11 novel HDAC4 inhibitor structures. All compounds designed were shown to outcompete in silico all parent compounds. This supports the utilization of the optimization guidelines developed here and presents 11 compounds that could be further investigated for their potential as a targeted glioma therapeutics.

## 2. Results and Discussion

### 2.1. Active Sites of HDAC4

The active site of HDAC4 consists of several His and Asp residues at the base of the gorge, which coordinates with the Zn^2+^ ion. Located on opposite sides of the gorge in the core of the active site are the two Phe-Phe motifs (Phe-812 and Phe-871). On the one side of the entrance of the gorge lies Asp-759 and Thr-760. The core of HDAC4 contains residues Phe-812, Phe-871, and His-842. These residues allow for π-π stacking in the gorge. The base of the active site includes residues His-802, His-803, Asp-840, Asp-934, and Arg-681 that are primarily involved in metal ion coordination with Zn^2+^ ion. HDAC4 is known to have a lower selectivity pocket (LSP) formed by the eight residues Pro-676, Glu-677, Arg-681, Pro-800, Glu-973, Gly-974, Gly-975, and His-976.

### 2.2. Docking Analysis of Original Cyclopropane Hydroxamic Acid Derivatives

**Figure 1:**
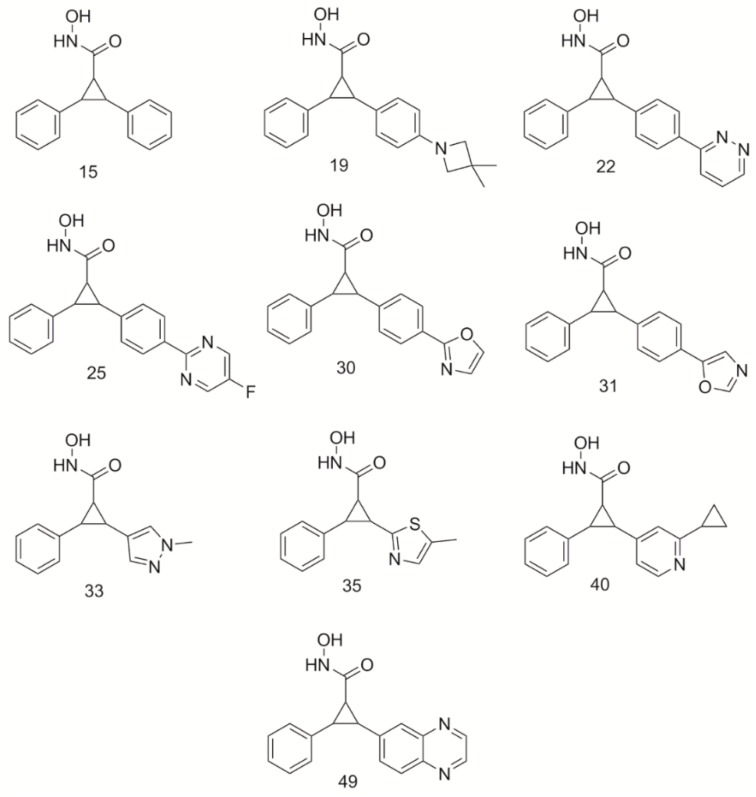
Structures of cyclopropane derivatives from Burli, 2013 [16] used as an original dataset.

Ten compounds (Figure 1) from the Burli, 2013 ranging in IC_50_ from 0.2 to 0.5 µM were selected as the initial dataset for analysis of binding interactions. All ten compounds were docked into HDAC4. Docking scores for each compound both in receptors are shown in Table 1. The modes of binding in HDAC4 were compared to determine common interactions and to evaluate new interactions that could be harnessed for enhancing HDAC4 potency. Each cyclopropane derivative produced similar interactions within the HDAC4 receptor. A mode of binding having bidentate coordination of the Zn^2+^ atom with the hydroxamate tail was produced, which is consistent with crystal structures of other hydroxamic acid HDAC inhibitors [2,21,23,24]. Similar to panobinostat, the cyclopropane derivatives were shown to be able to participate in π-π stacking interactions within the core of the active site between the residues Phe-812 and Phe-871 lining the gorge [25]. The benzene ring was shown to be positioned within the LSP of HDAC4. The observed poses and interactions were all consistent with the crystal structure of HDAC4 complexed with compound 25 and compound 31 [16]. 

### 2.3. Aliphatic Moieties in the Lower Selectivity Pocket Improve Binding Affinity 

The selectivity of the HDAC4 inhibitors is predicated on the benzene ring accessing the LSP [16]. The designed compounds from Burli, 2013, were shown to position the aromatic benzyl ring in the LSP. This pocket is primarily hydrophobic and thus, the placement of the benzene ring interacts well in this space. Hydrophobic interactions can, however, be created with both aliphatic and aromatic moieties. It was hypothesized that both types of interactions might be useful in binding to the HDAC4 receptor, however, one might be favored resulting in stronger binding. Therefore, this work sought to determine if a preference existed for aromatic or aliphatic hydrophobic moieties in the LSP of HDAC4. Nine derivatives (S25d-01, S25d-02, S25d-03, S25d-04, S25d-05, S25d-06, S25d-07, S25d-08, S25d-09) of compound 25 were created by replacing the benzyl ring with an aliphatic substituent (Supplemental Figure S1). These compounds were docked into HDAC4 to determine binding mode and potency. Docking scores for these derivatives are shown in Table 2.

All compounds having aliphatic moieties were shown to have improved binding affinities (an increase of 0.79–2.95 in −log10(Kd) value) to HDAC4 receptor compared to the parent compound 25 (docking score 8.00), which contains an aromatic phenyl ring that is positioned in the LSP. The mode of binding was still similar to the parent compound, having a bidentate interaction between the Zn^2+^ atom and the hydroxamate tail of all derivatives. The phenyl ring in the core of each derivative was found to form π-π stacking interactions within the core of the active site between the residues Phe-812 and Phe-871 lining the gorge. Compounds S25d-07 and S25d-09 had the greatest improvement in binding score, increasing affinity by at least 2.5 orders of magnitude. Compounds containing cyclized aliphatic moieties (S25d-06, S25d-04, S25d-08, S25d-02, S25d-01, S25d-05) also produced scores that were better than compound 25 enhancing binding affinity by at least 0.8 orders of magnitude. Compound S25d-03 was shown to enhance binding affinities to HDAC4 by 0.79 orders of magnitude, similar to S25d-05. 

**Figure 2:**
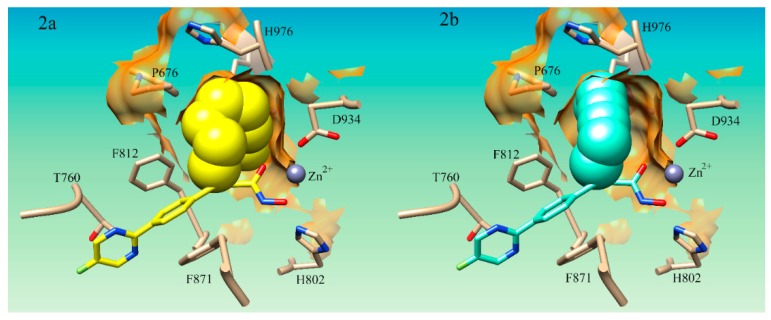
Docking pose of aliphatic compounds S25d-07 and S25d-03 in HDAC4 active site. (**2a**) Compound S25d-07 (yellow) in HDAC4 active site; (**2b**)Compound S25d-03 (light cyan) in HDAC4 active site. The aliphatic substituent in lower selectivity pocket is shown in sphere conformation to demonstrate the flexible linker’s ability to access more hydrophobic interactions in the lower selectivity pocket (LSP) compared to the more rigid structure of S25d-03.

It was noted that S25d-07 and S25d-09 had linear aliphatic R-groups which could have significant conformational flexibility. Both S25d-07 and S25d-09 had linear aliphatic tails that were shown to wrap around the internal pocket and fill more of the space in the lower binding pocket (Figure 2a). The middle group of compounds S25d-06, S25d-04, S25d-08, S25d-02, S25d-01, and S25d-05 all had cyclized aliphatic R-groups which restricts some of the conformational flexibility of the substituent. Compound S25d-03 which had the lowest improvement in binding affinity contained a triple bond creating the most rigid system evaluated. Compound S25d-05 was similar in binding score to S25d-03 and the lowest scored cyclized intermediate. This compound has a diene in the ring system and thus, had the largest degree of rigidity of the cyclized R-groups tested. In addition to the rigidity, the methyl group and the isopropyl group on opposite sides of the ring create steric clashing in the pocket, also reducing the binding affinity.

Accessing more surface area of the hydrophobic LSP by using flexible aliphatic groups enhances binding affinity and could potentially increase the selectivity of compounds for HDAC4. The flexibility of the R-groups in the S25d-07 (Figure 2a) and S25d-09 compounds would allow them to make more contact in the pocket. The rigid triple bond R-group in compound S25d-03 (Figure 2b) forced the aliphatic moiety to position itself primarily in the center of the pocket prohibiting a number of hydrophobic interactions. Flexibility in the substituent targeting the LSP assists in capitalizing on the most hydrophobic interactions possible. Taken together, these results demonstrate that aromaticity is not preferred in the LSP as the aliphatic compounds improved binding affinity compared to the aromatic phenyl ring. This analysis has not been explored by any of the studies on cyclopropane derivatives for HDAC4 which we could find.

### 2.4. Molecular Interactions on the Rim of the HDAC4 Active Site Improve Binding

HDAC4 inhibitor compounds developed up to this point have not been shown to have any direct interaction with Asp-759 [16,21,22] which sits on the rim of the opening to the active site. This residue creates a negative-charge region that could participate in electrostatic interactions and could permit hydrogen bonding. Thus, we hypothesized that strong electrostatic or hydrogen bonding interactions with the Asp-759 residue could be developed and that they would provide enhanced potency. Using nine derivatives (S15d-02, S15d-03, S15d-04, S15d-05, S15d-06, S15d-07, S15d-08, S15d-12, and S15d-13) of compound 15 (Supplementary Figure S2) the effect of electrostatic interactions with Asp-759 were evaluated. Compounds were designed to have similar features to the amino acids lysine and histidine. The effect of ortho, meta, and para positioning of the lysine-like substitutes on binding affinity was also evaluated. In some compounds, a second benzene creating a biphenyl system was introduced. Ortho, meta, and para versions of these compounds were evaluated as well. Lastly, we evaluated the effect of the length of the lysine-like substituent on compounds having zero, one, two, three, or four methylene linkers between the cyclopropane ring and the cationic nitrogen which were designed. A docking score for these compounds is listed in Table 3. 

**Figure 3:**
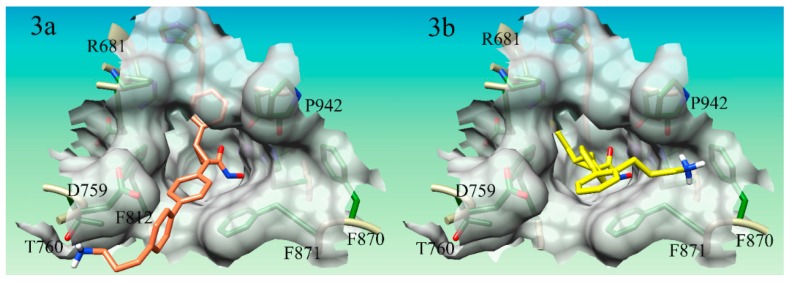
Docking pose of compounds S15d-06 and S15d-07 in HDAC4. (**3a**) Compound S15d-06 (salmon) in HDAC4 active site. Cationic nitrogen on the derivative is shown hydrogen bonding with T670 on the rim of the active site; (**3b**) Compound S15d-07 (yellow) in HDAC4 active site. The cationic nitrogen is shown forming a cation-pi interaction with F870 on the rim of the active site.

Seven of the nine compounds evaluated were shown to improve the binding affinity to the parent compound 15, having a binding score of 7.43, to HDAC4. Compound S15d-06 (Figure 3a) enhanced binding the most, improving the docking score of compound 15 from 7.43 to 10.15. Compounds S15d-06, S15d-04, S15d-07, S15d-03, and S15d-02, all increased the binding affinity by at least 1.6 orders of magnitude. Compounds S15d-12 and S15d-13 were shown to reduce binding affinity to HDAC4 than compound 15 from 7.43 to 6.48 and 7.05, respectively. Compound S15d-12 reduced binding affinity by at least one order of magnitude. It was shown that compounds having the biphenyl system generally produced stronger binding to HDAC4 than the single phenyl ring system, however, the difference was small, with one notable exception: the para-substituted compounds (S15d-06 and S15d-08). The para-substituted biphenyl ring compound S15d-06 produced a docking score of 10.17 while the para-substituted singular phenyl ring compound S15d-08 derivative only produced a score of 8.72. 

Like the derivatives of compound 25 mentioned previously, the derivatives of compound 15 were shown to have a mode of binding similar to the parent compound; encompassing a bidentate interaction between the Zn^2+^ atom and the hydroxamate tail in addition to the phenyl rings of each derivative forming a π-π stacking interaction within the core of the active site between the residues Phe-812 and Phe-871 lining the gorge. Two compounds had clear electrostatic interactions with Asp-759 (compounds S15d-13 and S15d-12) and two compounds positioned the cationic nitrogen in a negatively charged surface groove made by the Asp-759 (compounds S15d-06, S15d-08). S15d-12 and S15d-13 formed clear electrostatic interactions with Asp-759, however, both of these compounds produced lower docking scores (6.48 and 7.05, respectively) than the parent compound 15 (docking score 7.43). S15d-12 and S15d-13 also formed a cation-π interaction with Phe-871. Compounds S15d-06 and S15d-08 produced docking scores of 10.17 and 8.72, respectively, which were higher than the parent compound 15 (docking score of 7.43). The lysine-like substituent of these two compounds wraps around the electronegative surface groove in HDAC4. This conformation was additionally stabilized by a hydrogen bond between one of the polar hydrogens on the cationic nitrogen atom Thr-760 (Figure 3a). This Thr-760 hydrogen bond is a novel interaction that has not been utilized in HDAC4 inhibitor design. 

The modes of binding for the derivatives did produce some unanticipated interactions. Several of the compounds designed in this dataset did not produce any interactions with the Asp-759 as expected, however, some novel interactions for HDAC4 inhibitors supporting binding affinity were observed. A cation-π interaction between compounds S15d-03, S15d-05, and S15d-07 (Figure 3b) with residue Phe-870 was observed. S15d-03 was designed to have only one phenyl ring in the core structure, while S15d-05 and S15d-07 both had biphenyl ring systems in the core of the structure. Both S15d-03 and S15d-07 had substituents in the ortho position, while S15d-05 was meta substituted. All three compounds had three methylene linkers between the ring system and the cationic nitrogen. The ortho/meta positioning of the lysine-like substituent is already angled back toward the inside of the gorge, unlike the para-substituted compounds that are pointed out of the gorge. Thus, the angled meta and ortho compounds were generally shown to favor interactions on the side of the gorge opposite the Asp-759, as the conformation of these structures makes accessing cation-π interaction with Phe-870 more facile. This novel cation-π interaction with Phe-870 to the best of our knowledge has not been utilized in the design of HDAC4 inhibitors developed so far. In addition to this novel cation-π interaction, the cationic nitrogen is also shown to hydrogen bond to the backbone of Pro-942. These interactions could purposefully be included in ongoing efforts to optimize the binding affinity of HDAC4 inhibitors. 

Taken together, utilization of positively charged substituents to target the Asp-759 on the rim of the active site was not as predictable as thought, however, useful interactions at the surface were still uncovered. Compounds having the cationic nitrogen did significantly increase the binding affinity overall pointing to a useful design tool for HDAC4 inhibitors in addition to several other novel molecular interactions of interest: cation-π interactions with Phe-870 or Phe-871, hydrogen bonding with Thr-760 and the backbone of Pro-842. 

### 2.5. Heteroaromatic Rings in the Lower Selectivity Pocket

Heteroaromatic rings can provide opportunities for π-stacking, hydrophobic interactions, hydrogen bonding, and even electrostatic interactions. Most work on cyclopropane hydroxamic acid derivatives has utilized the phenyl ring as the main substituent to access the LSP [16,21,22]. Recent work has expanded this to rings having a single nitrogen atom [22]. This work further expanded this investigation to rings having diazine compounds, indole rings, and large aromatic substituents. To determine the impact of heteroaromatic rings in the LSP, a total of nine derivatives (S22d-07, S22d-08, S22d-09, S31d-01, S31d-02, S31d-04, S31d-05, S31d-06, S31d-07) were designed; three were derivatives of compound 22 and six were derivatives of compound 31. The phenyl ring that accesses the LSP was substituted with a variety of different heteroaromatic ring structures to determine any impact on binding affinity. The impact of the number of linkers between the cyclopropane ring and the aromatic substituent was also explored. The derivatives of compounds 31 and 22 are shown in Supplementary Figure S3, and the docking scores produced are listed in Table 4.

Of the nine compounds evaluated, only S31d-06 and S31d-07 having docking scores of 9.02 and 9.41, respectively, were shown to produce docking scores better than the parent compound 31, which produced a docking score of 8.22. Compound S31d-06 was designed to have an indole substituent to reside in the LSP, while S31d-07 was designed to have a diazine ring in the LSP. Both of these compounds had two methylene linkers between the cyclopropane ring and the aromatic substituent. As anticipated, the mode of binding for these two derivatives adopted resulted in the indole and diazine ring substituent being placed in the LSP. While these substituents are fairly large, it was discovered that the two methylene linkers between the cyclopropane ring and the aromatic ring in S31d-06 and S31d-07 allow these substituents to have more conformational flexibility compared to the other derivatives designed. This conformational flexibility allows them to fill the LSP more effectively than the compounds having one or no linkers between the cyclopropane ring and the aromatic substituent. This result is consistent with our previous study using various aliphatic substituents in the LSP, further suggesting the conformational flexibility has a significant impact on accessing the LSP effectively. 

It was noted that many of the compounds had the phenyl-oxazole ring positioned in the LSP instead of the desired substituent designed for this purpose. Compounds S31d-01, S31d-02, and S31d-04, were shown to dock the phenyl-oxazole ring system, which typically binds in the core of the gorge between Phe-812 and Phe-871 in the LSP instead of the aromatic substituent designed for the LSP. To explain this result, the length of the substituent from the cyclopropane ring to the end of the substituent was measured and compared to the length of the phenyl-oxazole ring system. Derivatives S31d-01, S31d-02, and S31d-04 were found to be 10.1 Å, 8.6 Å, and 7.7 Å, respectively, while the phenyl-oxazole system was found to be 7.9 Å. Thus, the designed substituents for S31d-01 and S31d-02 were significantly longer than the phenyl-oxazole ring system, resulting in the phenyl-oxazole ring being preferentially placed in the LSP. It is noteworthy that compound S31d-06 has a substituent that is approximately 10.2 Å, yet interacts in the LSP well. When docked into the LSP, the linker rotates creating an overall distance of 3.6 Å between the cyclopropane ring and the furthest point of the substituent. Thus, while this derivative is longer in size compared to the phenyl-oxazole ring system, the flexibility of the linker region permits it to adopt a shorter conformation overall allowing for better packing in the LSP than S31d-01 and S31d-02. While S31d-04 was not longer than the phenyl-oxazole ring system, this compound did have an alcohol substituent. Previous work demonstrated that polar substituents in the LSP were more favorable [22]. This prior work focused on six-membered rings having a polar substituent but did not use indole variations in the study [22]. While the S31d-04 derivative has polar OH as a substituent, the ring is an indole variant, thus spatial positioning of the OH substituents would be different spatially than that observed on a six-member ring. Thus, it is hypothesized, that due to the different placement of the polar group on the indole, the phenyl-oxazole ring system was preferred in HDAC4′s LSP. Even though the phenyl-oxazole ring system was preferred over the indole type ring system, S31d-04 decreased binding affinity significantly. 

Compounds S31d-05, S31d-06, and S31d-07 did not position the phenyl-oxazole ring in the pocket. In compound S31d-05, the phenyl-oxazole ring system is also not positioned to effectively participate in the double π-π stacking interactions with Phe-812 and Phe-871 in the gorge. Rather it adopts a less favorable edge-to-edge T-shaped interaction with both residues, thus lowering the binding score. While the derivatives of compound 31 mostly decreased affinity of the compound for HDAC4, they were all shown to have a bidentate interaction with the Zn^2+^ ion and the hydroxamate acid tail, unlike the derivatives of compound 22. Derivatives of compound 22 have an azo group in the outermost phenyl aromatic ring. This ring would typically interact in the core with the Phe-812 and Phe-871 forming π-π stacking interactions. The azo group in the core phenyl-diazine structure, however, was positioned to coordinate with the Zn^2+^ ion in a bidentate fashion. These compounds positioned the six-membered nitrogen-containing heterocycle in the aromatic pocket on the surface of the gorge created by Phe-870 and Phe-871. The nitrogen heterocycle forms a parallel-displaced stacking interaction with Phe-871 and a T-shaped interaction with Phe-870. 

Analysis of the derivatives containing heterocycles designed to interact in the LSP revealed several different modes of binding compared to the parent compounds. Several considerations can be garnered from this study for structural design. First, the LSP has a size limitation to what types of structures can be placed in the pocket, but this size can be accommodated if there is flexibility in the substituent. It maybe possible to use non-hydroxamic acid derivatives of a cyclopropane compound to bind to HDAC4. The benzamide HDAC inhibitors are an example of HDAC inhibitors that coordinate to the Zn^2+^ atom using at least one nitrogen atom [23,26,27]. The derivatives using the bidentate nitrogen system to complex with the Zn^2+^ ion also revealed that a parallel-displaced stacking interaction with Phe-871 and a T-shaped interaction with Phe-870 can be accessed simultaneously on HDAC4. No derivatives were specifically designed to target this type of interaction but another study to interrogate the importance of this interaction would be worthwhile.

### 2.6. Heteroatoms in the Innermost Aromatic Ring of the Scaffold

HDAC receptors are known to have a Phe-Phe motif in the core of the active site which is why it has been found that panobinostat outperforms vorinostat in potency [28]. The benzene core of panobinostat can participate in π-π stacking interactions with the Phe-Phe motif, while vorinostat having a purely aliphatic linker region cannot. Currently, the inclusion of heteroaromatic rings used in hydroxamic acid derivatives for HDAC inhibitors development has also been evaluated [16,21,22,25]. In studies focused on HDAC4, tandem aromatic rings cores have also been explored, evaluating ring systems combining either two 6-membered aromatic rings, or an aromatic 6-membered and aromatic 5-membered bound to each other [16,21,22]. These structures which were designed to interact with the Phe-812 and Phe-871 residues in the core of the HDAC4 active site, however, only evaluated heteroatoms in the outermost ring of the tandem ring system (ring closest to the entrance to the active site). The influence of heteroatom inclusion in the innermost ring (ring which is directly attached to the cyclopropane ring) has not been explored. A heteroaromatic ring in the innermost position might allow for more hydrogen bonding interactions with the histidineresidues in the active site [25]. Thus, derivatives of compounds 22, 25, 30, and 31 (Supplementary Figure S4) were designed to determine the impact of heteroatom inclusion in the innermost ring of cyclopropane derivatives on HDAC4 inhibitor potency. The innermost phenyl ring was substituted with one of three diazine structures. Twelve derivatives in total were developed (S22d-01, S22d-02, S22d-03, S25d-15, S25d-16, S25d-17, S30d-04, S30d-05, S30d-06, S31d-08, S31d-10, and S31d-11). The docking scores for each compound are shown in Table 5.

Results showed that each individual parent compound was affected differently by the inclusion of a diazine ring in the innermost structure. Derivatives of compound 25 showed significant improvement in overall binding affinity, having an average improvement of 1.49 in the docking score. Derivatives of compound 22 had produced a small average increase of about 0.11 in the docking score. Derivatives of compounds 30 and 31 both resulted in decreasing the affinity to the HDAC4 receptor. The overall decrease in affinity was 0.19 for compound 30 derivatives and slightly more moderate for compound 31 derivatives which produced an average decrease of 0.37 in binding affinity. Differences less than 1 in the −log Kd value docking scores would result in minor differences in compound binding affinity, therefore, the derivatives of compounds 22, 30, and 31 would have a binding affinity to HDAC4 that was similar to the respective parent compounds. Derivatives of compound 25, however, would be predicted to have a significantly better binding affinity than the parent compound. 

Structurally, derivatives of compounds 30 and 31 have an oxazole ring as the outermost ring, while derivatives of compounds 22 and 25 have a diazine ring as the outermost ring in the compound. The tandem diazine-diazine ring system performed better than the oxazole-diazine ring combination. This suggests that heteroatoms in the innermost ring can aid in enhancing binding affinity, however, other factors will be important to evaluate, such as the specific type of aromatic-aromatic ring system. The results presented here can only speak to the data demonstrating that a diazine-diazine combination can work well, however, it is not guaranteed that a large improvement can be garnered as the derivatives of compound 22 did not produce a large improvement in binding. A DFT study evaluating more tandem aromatic rings systems having a more diverse set of combinations would be ideal to interrogate this observation further.

### 2.7. Hybrid Compounds 

**Figure 4:**
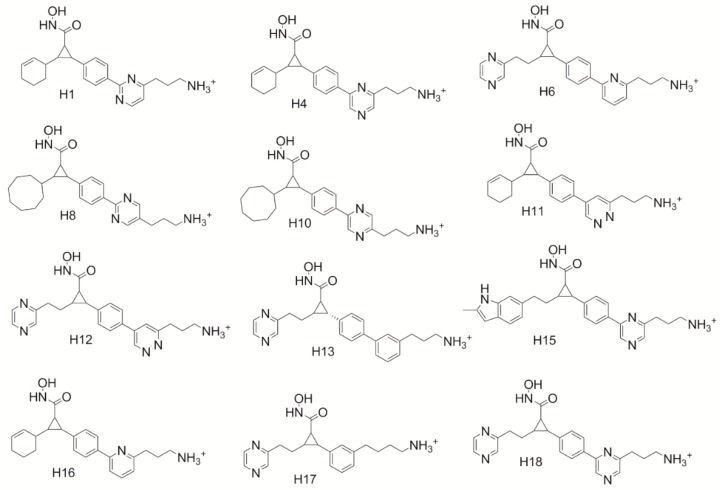
Structure of our designed hybrid compounds having optimized molecular features to interact.

Based on these tools, hybrid compounds were designed (Figure 4) to evaluate the optimization guidelines garnered from the study. It was hypothesized that a hybrid between S25d-07 and S15d-06 would garner the most significant increase in binding affinity, however, the log *p* of this compound was 4.88 due to the introduction of two very large hydrophobic substituents. Thus, due to concerns about the ability of the initial set of designed hybrid compounds to be drug-like, Lipinski’s rule of five was used to determine which compounds would be the best candidates for further analysis [29]. A second version of the hybrid compounds which incorporated nitrogen atoms in the core ring structure was designed to improve the drug-likeness of the compounds. Hybrid compounds using S31d-07 and S15d-06 were also envisioned as good candidates. Unfortunately, these compounds were unable to be evaluated by the docking software Sybyl-X. A total of eleven novel cyclopropane hydroxamic acid compounds were, however, successfully evaluated in the HDAC4 receptor to predict the binding affinity. Docking scores for these compounds are listed in Table 6.

**Figure 5:**
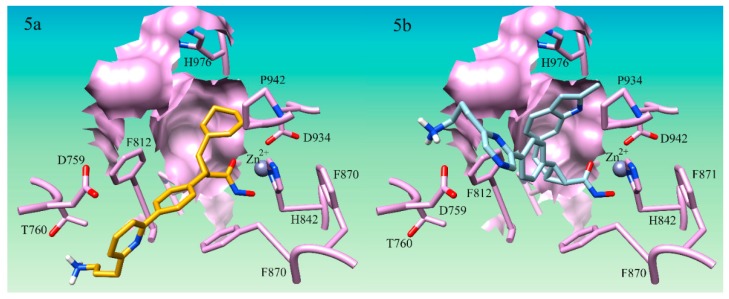
Docking pose of H16 and H15 in HDAC4 active site. (**5a**) Docking pose of compound H16 (gold) in HDAC4 active site Hydrogen bond interaction between cationic nitrogen and T760 is shown; (**5b**) Docking pose of compound S25d-03 (light blue) in HDAC4 active site. Cationic nitrogen is shown forming an electrostatic interaction with D759 in the active site.

All eleven hybrid compounds were shown to outcompete all of the original parent compounds used in this study. H16 (Figure 5) was shown to have a binding score of 12.35 in HDAC4, outperforming compound 40, the top-ranked cyclopropane hydroxamic acid derivative developed by Burli, 2013 by 3.53 and compound 15 the lowest-ranked parent compound used by 4.92. This suggests that this hybrid compound is predicted to be almost 1000-fold better than the original cyclopropane derivatives. H11 showed the least improvement in binding overall in the HDAC4 receptor having a score of 9.53. H11 is still, however, better than all the parent cyclopropane derivatives, thus still a potentially potent HDAC4 inhibitor. The hybrid compounds accessed the predicted molecular interactions discovered to enhance binding in the study here. Only two compounds, H12 and H15, produced new modes of binding that were not anticipated. H15 (Figure 5b) was not found to hydrogen bond to Thr-760 as expected, but was found only to participate in an electrostatic interaction on the side of the Asp-759 opposite the Thr-760 hydroxyl group. Remarkably, this is how it was envisioned that the inclusion of the cationic lysine-like derivatives would preferentially bind in our first study designed to target Asp-759. H12 was also not found to hydrogen bond to Thr-760 but was shown to position to the azo group in the ring next to Asp-759. Even though H12 and H15 produced new modes of binding, they still were shown to enhance the binding affinity of the HDAC4 cyclopropane inhibitor compounds. Therefore, it can be seen that the combination of multiple new molecular interactions identified through this work does reliably lead to an optimization in the predicted binding affinity supporting the utility of the design guidelines above.

It is noteworthy to mention that HDACi have been found to affect histone modifications [30,31,32,33,34]. HDAC4 is specifically known to dictate demethlyation of H3K9 and HP1 disassociation in response to cardiac load variations [33]. It has been shown that increased levels of HDAC4 decrease H3K14 acetylation, leading to an increase in SUV39H1 methylase activity which results in the H3K9 di-methylation [35]. This suggests that the proposed inhibitors would reverse these effects as they would disrupt HDAC4 ability to deacetylate H3K14. The impact of HDAC4 specific inhibitors and epigenetic histone modifications has not been reported specifically, however, we propose that the compounds designed here could open doors to expand on work in this area.

HDAC4, which is elevated in glioma cells, has been demonstrated to be essential for tumorigenesis of glioma. When HDAC4 was knocked down in U251, a human gliomablastoma cell line, the proliferation ability of the cells was significantly decreased [7]. When HDAC4 was overexpressed in U251 cells, the glioma cell’s invasive ability was enhanced. By inhibiting the expression of HDAC4 in U251 cells, the invasive ability was diminished [7]. These findings from previous research support the use of HDAC4 as a therapeutic target for glioma, as inhibition of this enzyme would result in decreased proliferation and invasiveness of this tumor type. The proposed inhibitors designed in this study are derivatives of a known class of HDAC4 inhibitors previously developed [16,21]. The selected template derivatives ranged from 0.54 µM to 0.02 µM inhibition of HDAC4 [16]. Thus, we propose that the developed compounds would be viable candidates to be pursued for glioma treatment due to the findings that cyclopropane hydroxamate acid derivatives are known to inhibit HDAC4 and that HDAC4 inhibition of glioma cells prevents proliferation and invasion of the tumor. 

## 3. Materials and Methods

### 3.1. Homology Modeling and Preparation of Receptors for Docking

The human HDAC4 receptor was imported from the PDB database into UCSF Chimera [35]. In this work, the HDAC4 crystal structure used as a receptor for docking analysis was 4CBT [16]. For docking simulations in this study, only the A chain of HDAC4 was used. The hydrophobic, hydrogen bond donor and acceptor regions of the active site were defined in the protomol using the SFXC protocol. The rest of the HDAC4 docking receptor was then prepared. In all receptor preparations, the Zn^2+^ ion located within the active site of HDAC4. 

### 3.2. Preparation of Ligands for Docking

In this study, a total of 60 cyclopropane hydroxamic acid structures were analyzed. All structures were drawn in ChemDraw, then converted to 3D format in Marvin Sketch (Chem Axon: Budapest, Hungary). Following 2D to 3D conversion, structures were initially minimized using an energy gradient optimization method. Full structural minimization was performed in UCSF Chimera (San Fransico, CA, USA), using steepest decent followed by conjugate gradient, to calculate total charges on the molecule and add hydrogens that were not explicitly defined in the 2D structure. All cyclopropane hydroxamic acid compounds were then pooled into one mol2 file which was used for docking. Using the Docking >1 parameter multiple conformers of each compound were created during the ligand structure preparation process in Sybyl-X 2.1. 

### 3.3. Docking Ligands into the Receptor

The Surflex-Dock Geom (SFXC) protocol was used for all docking simulations in order to predict each compound’s binding affinity to the HDAC receptors. The C-scoring method was used to calculate all predicted binding affinities and are reported as the −log10(Kd) value. A stronger binder is indicated by a larger −log10(Kd)value. Sybyl-X and UCSF Chimera programs were utilized to visualize and evaluate the binding interactions of the compounds in the HDAC receptors. UCSF Chimera was used to create the figures.

### 3.4. Structural Evaluation of HDAC Receptors

Characteristics of HDAC4 were evaluated to determine molecular interactions that could be accessed in and near the active site. An electrostatic potential map of HDAC4 was developed using UCSF Chimera and also utilized to evaluate binding preferences to docked substrates.

## 4. Conclusions

A novel set of molecular tools has been demonstrated as useful in the optimization of HDAC4-inhibitors. The most significant results validated that the aliphatic substituents, with an emphasis on substituents having some degree of flexibility to explore the LSP cavity, cation-π interactions with Phe-870 and Phe-871 at the entrance of the active site, hydrogen-bonding interactions between Thr-760, and hydrogen bonding interactions with the backbone of Pro-842 are novel molecular interactions that can successfully be targeted in the design of HDAC4 inhibitors to improve binding. In addition to these supportive features, it was demonstrated that using tandem heterocycles in the core of the structure used to interact with the Phe-Phe motif in HDAC4 can contribute to stronger binding, however, more work is needed to determine which type of tandem heterocycle ring systems are best. Lastly, it was determined that aromatic substituents targeting the LSP having heteroatoms in the structures could improve binding affinity when a linker was added to allow for the ring to access more of the LSP.

These new optimization tools have produced eleven novel cyclopropane hydroxamic acid compounds that outcompete all the parent cyclopropane hydroxamic acid derivatives evaluated. The most significant enhancement was garnered by hybrid compound H16 (Figure 5), showing an enhancement of 5.86 in binding affinity over the first generation cyclopropane derivative 15. Thus, by utilization of these design guidelines, potency can be enhanced for HDAC4 inhibitors. HDAC4 inhibitors could be an essential link to developing targeted treatment for glioma tumors or studying the role of HDAC4 in cancer research. This work proposes novel compounds for glioma and other disorders sensitive to HDAC4 expression and provides researchers with tools to promote maximal binding affinity to the HDAC4.

## Figures and Tables

**Table 1:** Docking scores of original cyclopropane derivatives in histone deacetylase 4 (HDAC4) and histone deacetylase 5 (HDAC5) receptors.

Compound	Inhibitor Strength	HDAC4 IC_50_ (µM)	HDAC4 Docking Score −log10 (Kd)
40	Strong	0.02	8.82
49	Strong	0.02	8.45
31	Strong	0.02	8.22
19	Moderate	0.33	8.17
25	Strong	0.05	8.00
30	Strong	0.05	7.98
22	Strong	0.08	7.73
15	Moderate	0.34	7.43
35	Moderate	0.10	7.17
33	Moderate	0.54	6.61
Average			7.86

**Table 2:** HDAC4 docking scores of cyclopropane derivatives having aliphatic moieties in the lower selectivity pocket.

Compound	Docking Score −log10 (Kd)
S25d-07	10.95
S25d-09	10.33
S25d-06	9.98
S25d-04	9.85
S25d-08	9.71
S25d-02	9.54
S25d-01	9.14
S25d-05	8.81
S25d-03	8.79
25	8.00

**Table 3:** HDAC4 Docking scores of cyclopropane derivatives compounds designed to have electrostatic interactions with Asp-759 residue on rim of HDAC4.

Compound	Docking Score −log10 (Kd)
S15d-06	10.17
S15d-04	9.75
S15d-05	9.68
S15d-07	9.39
S15d-03	9.25
S15d-02	9.01
S15d-08	8.72
15	7.43
S15d-13	7.05
S15d-12	6.48

**Table 4:** HDAC4 Docking scores of cyclopropane derivatives having heteroaromatic rings in the lower selectivity pocket.

Compound	Docking Score −log10 (Kd)
S31d-07	9.41
S31d-06	9.02
31	8.22
22	7.73
S22d-08 (*n*)	5.73
S22d-09 (*n*)	5.38
S31d-01	5.34
S31d-05	5.08
S22d-07	5.04
S31d-04	4.51
S31d-02	3.46

**Table 5:** HDAC4 docking scores of cyclopropane derivatives having heteroatoms in the innermost aromatic ring of the core of the scaffold.

Compound	Docking Score −log10 (Kd)
S25d-15	9.69
S25d-16	9.53
S25d-17	9.24
31	8.22
25	8.00
30	7.98
S31d-10	7.97
S31d-08	7.91
S22d-01	7.86
S22d-03	7.85
S30d-04	7.82
S30d-06	7.82
S22d-02	7.81
22	7.73
S30d-05	7.73
S31d-11	7.67

**Table 6:** Docking scores of our designed cyclopropane hybrid derivatives in HDAC4 and HDAC5 receptors.

Compound	HDAC4 Docking Score −log10(Kd)	Log *p*-Value
H16	12.35	2.07
H17	12.12	0.90
H1	11.58	1.19
H4	11.16	0.96
H6	10.73	3.72
H8	10.66	1.05
H12	10.66	2.77
H13	10.25	1.84
H15	9.99	1.88
H10	9.94	2.77
H11	9.53	2.86
40	8.82	2.72
49	8.45	2.14
31	8.22	2.07
25	8.00	3.76
30	7.98	2.39
22	7.73	3.34
15	7.43	2.97

## Supplementary Materials

Supplementary materials can be found at https://www.mdpi.com/1422-0067/21/1/219/s1.
