# Supplementary material for: In silico Design of Novel Histone Deacetylase 4 Inhibitors: Design Guidelines for Improved Binding Affinity
Source: Int J Mol Sci. 2019 Dec 28;21(1):219. doi: 10.3390/ijms21010219 (PMC6981887; doi:10.3390/ijms21010219)
Supplement: Supplementary file 1 [file ijms-21-00219-s001.pdf]

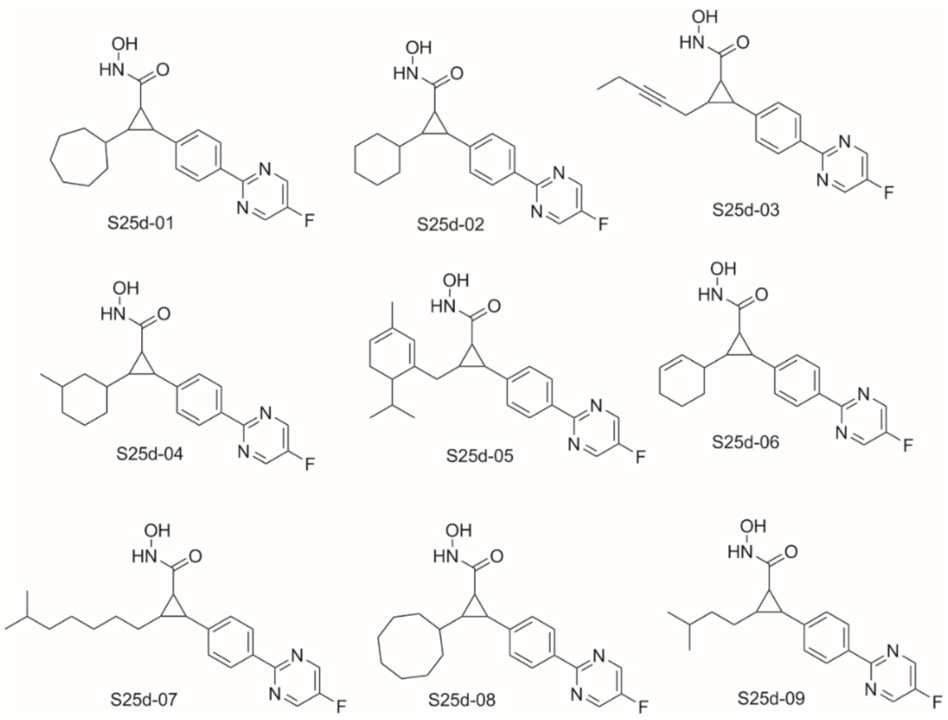

Figure S1. Structures of compound 25 derivatives having aliphatic moieties targeting the lower binding pocket in HDAC4.

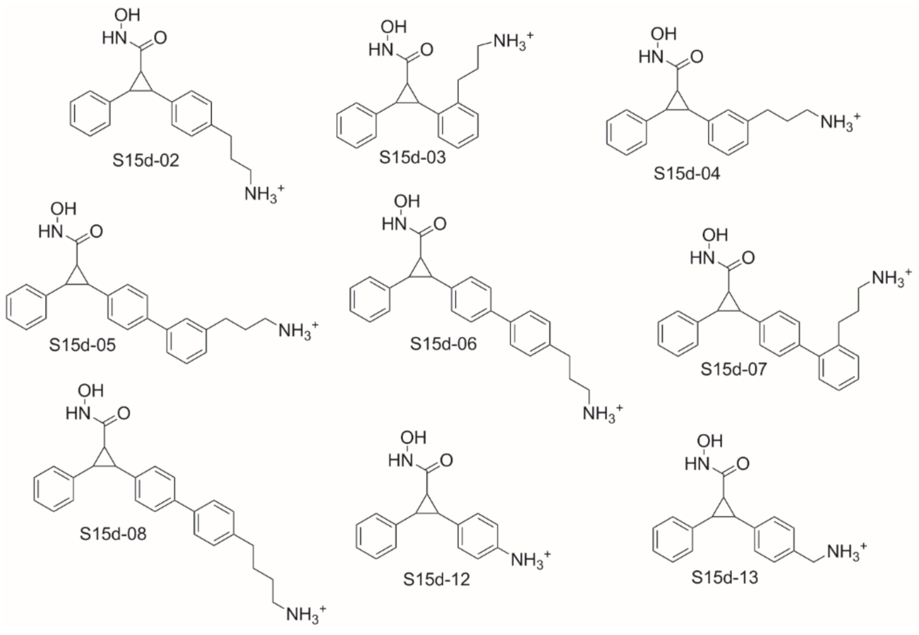

Figure S2. Structures of compound 15 derivatives having positively charge substituents designed to target Asp-759 in HDAC4.

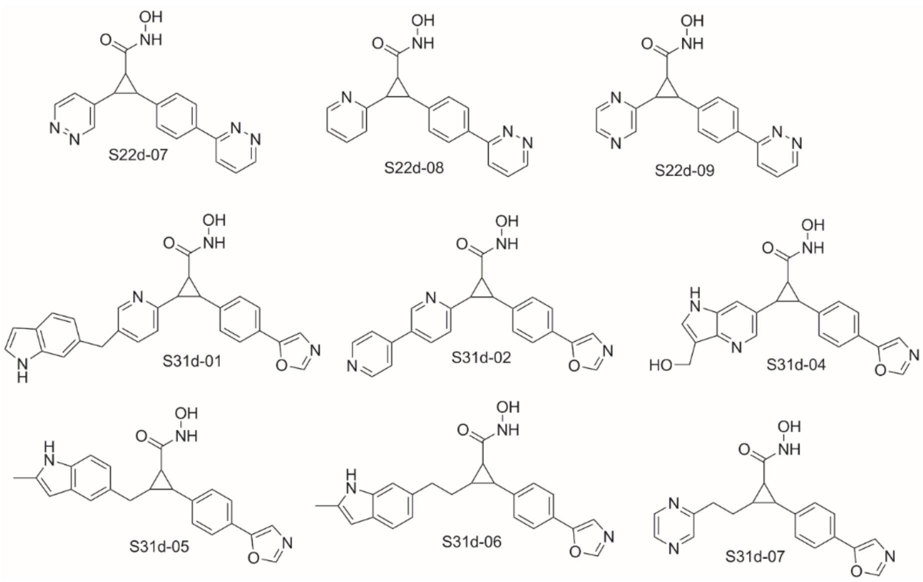

Figure S3. Structures of compound 31, 32, 22, and 25 derivatives having heteroatoms in the aromatic ring designed to target the lower selectivity pocket in HDAC4.

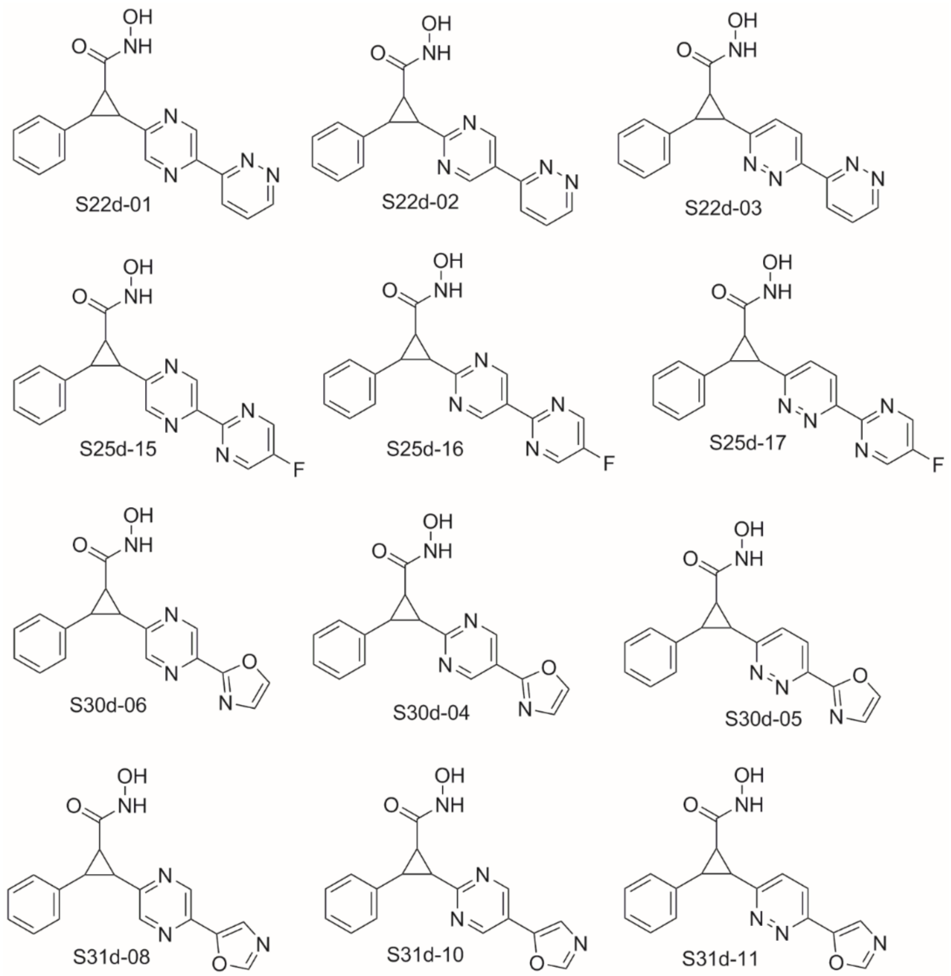

Figure S4. Structures of compound 31, 25, 22, and 30 derivatives having heteroatoms in the core aromatic ring designed to interact with Phe 812 and Phe 871 in HDAC4.
